# Best Dressed Test: A Study of the Covering Behavior of the Collector Urchin *Tripneustes gratilla*

**DOI:** 10.1371/journal.pone.0153581

**Published:** 2016-04-13

**Authors:** Morgan A. Ziegenhorn

**Affiliations:** Department of Integrative Biology, University of California, Berkeley, California, United States of America; Department of Agriculture and Water Resources, AUSTRALIA

## Abstract

Many sea urchin genera exhibit cryptic covering behaviors. One such behavior has been documented in the sea urchin *Tripneustes gratilla*, and previous studies have theorized that this behavior serves as protection from UV radiation. However, other hypotheses have been presented such as protection from predators or added weight to help *T*. *gratilla* resist strong currents. A field study was conducted in October-November 2015 in Moorea, French Polynesia to assess urchin covering behavior in natural habitats. The study found that urchins partially underneath rocks covered more, and with more algae, than urchins totally underneath rocks. To test if this behavior was driven by light intensity, a series of 30-minute experimental trials were run on 10 individuals in bright and dim conditions. Individuals were given red and clear plastic, and percent cover of each was recorded. These tests were repeated once fifty percent of spines had been removed from the urchin, in order to determine whether spine loss affects *T*. *gratilla* covering behavior. The study found that urchins had a distinct preference for cover that best protects them from UV radiation. Spine loss did not significantly affect urchin ability to cover, and urchins with removed spines still preferred opaque cover. Additionally, covering behavior was mapped onto a phylogeny of echinoderms to determine how it might have evolved. Understanding urchin covering behavior more fully is a step towards an understanding of the evolution of cryptic behavior across species.

## Introduction

Crypsis, the process of avoiding observation or detection by other species, is a widely seen phenomenon in the animal kingdom [1]. Many organisms including various mollusks, flies, and crabs exhibit cryptic behaviors such as camouflage and mimicry [2]. Crypsis can serve many functions including protection from predation, barriers against harmful environmental factors, or concealing species from prey while hunting.

In sea urchins (class Echinoidea), cryptic behavior involves using tube feet in conjunction with spines to hoist and secure materials to the aboral surface [3], or, in the case of floating materials, seizing objects directly with tube feet [4]. Though this behavior is exhibited by several different urchin species, it remains a poorly understood phenomenon [5], and reasons for covering are thought to differ between species. Some species, like *Stronglyocentrotus drobachiensis*, cover to a higher degree when exposed to wave surges [5], while in other species such as *Evichinus chloroticus* covering is mainly a form of food capture [6].

In the case of the urchin *Tripneustes gratilla*, commonly known as the “collector urchin”, many possible hypotheses for covering behavior have been explored, including protection from predators, protection from light exposure, and protection from strong currents [7]. In several studies, a correlation between light intensity and urchin cover was noted [5, 7], and it has been postulated that *Tripneustes* covering behavior is a form of protection from the sun [8]. This conclusion is bolstered by the urchin’s ability to sense and respond to light via photo-sensitive tube feet [9].

Previous studies have concluded that collector urchins relinquish cover in lower light conditions because darkness is a trigger for nocturnal foraging, during which the urchins are more mobile and weighing less is more energetically favorable [7]. However, little work has focused on preference between covering materials with regard to light intensity. If sunlight is the main factor that influences covering behavior, urchins should prefer materials that best shield them from light. A consideration of cover preferences would therefore provide insight into the role of light, leading to a better understanding of how and why this behavior evolved.

Though studies have examined the mechanism of attaching materials to the test and found urchin spines to be an important part of this process [3], no previous research has examined how covering ability is affected by spine loss. Spine loss is a common phenomenon in urchins, and can occur at very high levels when it is a symptom of disease. One such disease that remains undefined has been observed in Hawaiian reefs [10]. However, spine breakage and regrowth remains undocumented in *T*. *gratilla*, though it has been thoroughly detailed for other urchin species, and the mechanism of regrowth across echinoderms is well understood [11]. Spine breakage most likely occurs from urchins being tossed by strong currents or from non-lethal interactions with predators such as pufferfish and humans that try to pry them out from under rocks. Understanding how spine loss affects important behaviors in urchins such as covering will aid in determining the likelihood that injured urchins are able to survive in their environment until their spines regrow.

The overall goal of this study was to characterize (1) how covering behavior in the urchin *T*. *gratilla* is represented in the field, (2) to what extent covering behavior is affected by differing light conditions, (3) how covering behavior is affected by spine loss, and (4) how covering behavior evolved in echinoderms. A field study was conducted to understand the influence of habitat, while the influence of cover type, light conditions, and spine loss was examined in the experimental study. Covering behavior was mapped onto a phylogeny of echinoderms to visualize how it most likely evolved. The hypotheses were (1) that covering behavior in the field would be linked to location on rocks, as urchins already protected from light by being underneath coral would not need to cover themselves, and no preference for covering materials was expected, (2) that there would be a statistically significant preference for opaque covering materials in bright light conditions, but not in dim light conditions where danger from light exposure was not a serious threat, (3) that this trend would remain unchanged once spines were removed, as covering behavior was expected to be related to light intensity, and not urchin defense, but with a slightly reduced covering ability, and (4) that urchin genera that cover themselves would be more closely related.

## Methods

**Fig 1:**
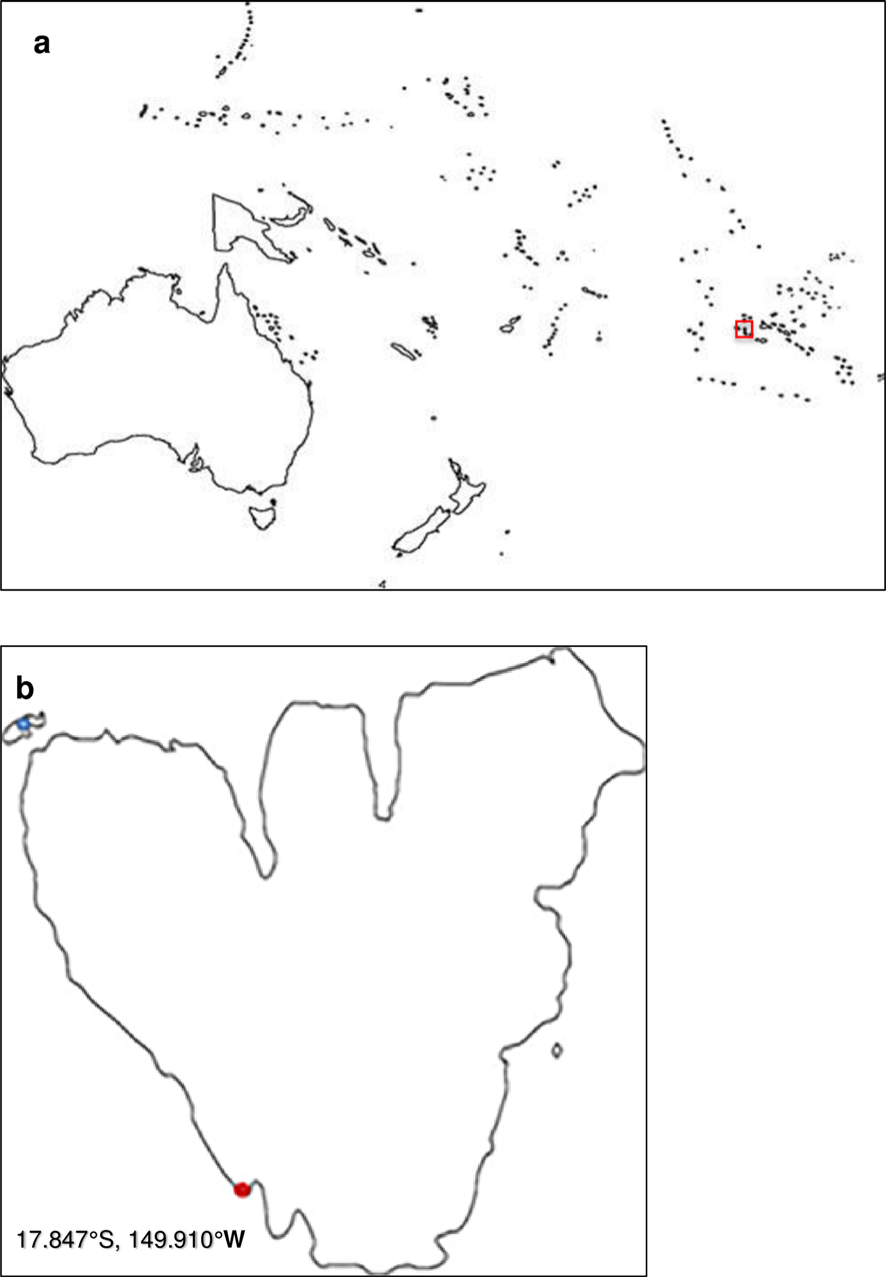
Maps of Moorea. (a) Map of the South Pacific, with the location (red box), latitude, and longitude of Moorea, French Polynesia noted. (b) Map of Moorea showing the field site between the motus Tiahura and Fareone (blue dot) and collection spot at Haapiti (red dot).

The study was performed at Gump Station in Moorea, French Polynesia (Fig 1A) in October and November 2015. Two sites were chosen, motu Tiahura and Fareone (Marine Protected Area), and Haapiti (an unprotected area) (Fig 1B). Urchins were collected at Haapiti since it was public land. The urchin *T*. *gratilla* is not an endangered or protected species, so no restrictions are in place to prevent testing on individuals of this species.

### Field study

Field surveys took place in the channel between the motus Tiahura and Fareone on the northwest side of the island Moorea, French Polynesia once a week from October 12, 2015 to November 10, 2015 (Fig 1B). A 30 by 50 meter site was surveyed by choosing 15 coral rocks on which *T*. *gratilla* urchins were seen. Each rock was thoroughly examined and the following data collected: location of urchin on rock (partially or totally underneath), and percent cover of algae and coral rock/shells on urchins. Percent cover was estimated by visually examining urchins in the field and estimating what percentage of their aboral side was covered by foreign materials (i.e. algae on a quarter of an urchin’s aboral side would have been recorded as 25% algal cover). Possible replication of some rocks in subsequent weeks was considered insignificant due to the assumption that, since urchins are not sessile, surveying the same rocks would not necessarily mean surveying the same urchins.

### Experimental study

Experiments were conducted at the Gump Station in Cooks Bay, Moorea, French Polynesia from October 16, 2015 to November 9, 2015. Ten *T*. *gratilla* urchins were collected from the reef at Haapiti on the southwestern side of Moorea (Fig 1B). As the detected population at Haapiti was small, multiple tests were run on this same small sample of urchins. Repetition of experiments with the same individual was not ideal, but was controlled for as much as possible by the use of proper statistical tests. However, it remains possible that small sample size caused some margin of error in the results of this study.

Urchins varied in size from 6.5 to 8.5 cm in test diameter. Urchins were kept in a tank with constant seawater flow from Cook’s Bay and fed a variety of macroalgal species, most notably *Sargassum sp*. and *Turbinaria sp*. Urchins were distinguished from one another via morphological characteristics such as test size, tube feet coloration, and spine coloration. A detailed description was recorded for each urchin and was used from then on to distinguish urchins from one another.

#### Pre-spine removal

**Fig 2:**
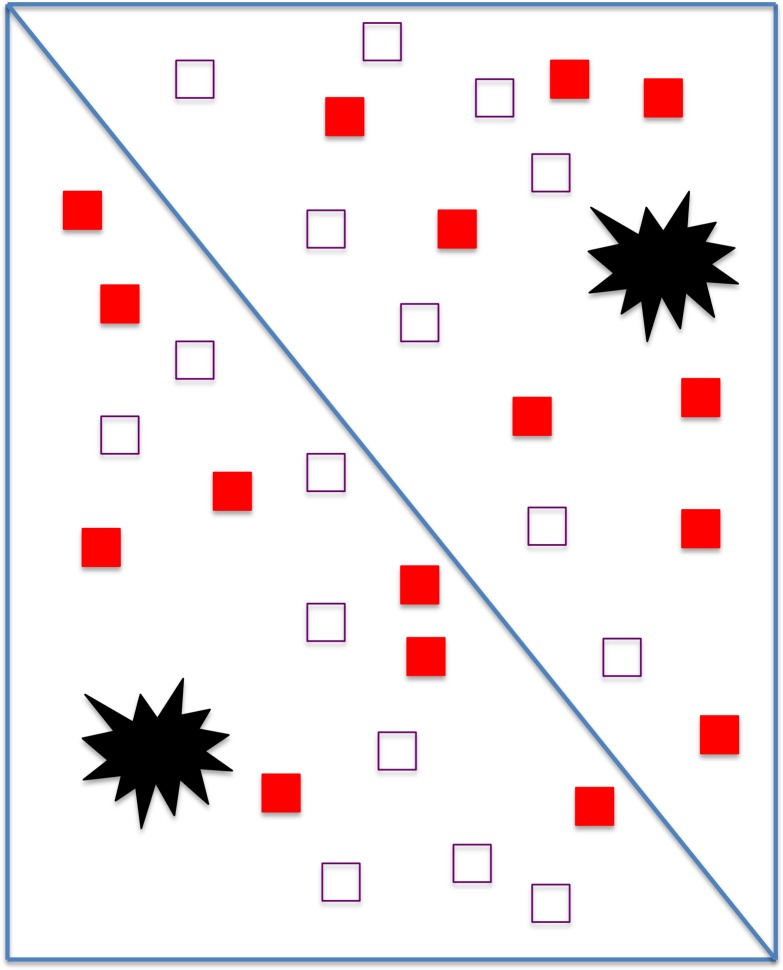
Experimental study set up. Diagram of urchin test setup, depicting the tub divided in two, the two urchins (in black) and the two cover types. Red plastic cover is represented by red rectangles, and clear cover is represented by clear rectangles with purple borders.

The experimental study involved testing urchins in two light conditions: bright sunlight and dim sunlight, and observing their covering behavior. For the bright light tests, two urchins were placed in one tub in direct sunlight with no available shade (Fig 2). This tub was divided in half so urchins could not reach each other. All cover was removed from the urchins prior to each test, and they were presented with eight pieces of hard red plastic and eight pieces of hard clear plastic. Red plastic was considered opaque in comparison to the clear plastic because it transmitted less UV light. The pieces of plastic were all of area 1–3 square centimeters. Percent cover was estimated by visual determination of the percentage of the urchin’s aboral side covered by each plastic type. Counting pieces of plastic covering the urchins was not used as a metric because not all urchins were the same size, and pieces of plastic attached to the urchins occasionally overlapped. This estimation was taken every minute for the first ten minutes, and then every five minutes for the second twenty minutes of the thirty minute experiment. The plastic covers were then removed from the urchins and the urchins returned to the larger tank.

This test was repeated on the urchins in the dim light condition after a rest period of at least forty-five minutes. The dim light condition was characterized by tests performed during daylight hours, outside, in complete shade. The temperature of the water in the tub was that of the sea water in Cook’s Bay (26 degrees Celsius), and water was replaced if it felt noticeably warmer to the touch. Six urchins were tested one day, and four the next. This cycle was repeated five times for a total of 10 tests (five bright and five dim) per urchin. No urchin went through more than one cycle of tests (one bright and one dim) in a single day. To control for the fact that urchins going through a second test in one day might be fatigued and cover less because of this, urchins were tested in dim light first some days and in bright light first on others.

#### Post-spine removal

The following week, spines were removed from the urchin’s test in order to test the effect of spine loss on covering behavior. Spines were clipped as close to the test as possible, and care was taken to not remove tube feet. Spines were removed over half the urchin in a semicircle that included both oral and aboral sides. Urchins were put through a dim light test of the same procedure as explained above directly after their spines were removed. After 2–3 hours, they were again subjected to bright light conditions. These tests were done in accordance with the method above a total of five times for each urchin, with every other day being a rest day for the individuals tested on the day prior. After the conclusion of these tests, urchins were returned to the reef off the Gump Station at Cook’s Bay. Urchins were not returned to Haapiti because of a lack of available transport.

### Phylogeny

The evolution of covering behavior in sea urchins was examined using an existing phylogeny of echinoderms [12]. Urchin genera from the phylogeny presented by Littlewood and Smith were searched on the internet both through images and articles to determine if they exhibited covering behaviors. This information was mapped onto the phylogeny using Mesquite [13] by creating a character matrix of all genera included in the phylogeny in which covering behavior was given a value of one, and no covering behavior a value of zero. This character matrix was analyzed and plotted onto the given phylogeny using the “Parsimony Ancestral States” option within the “Trace Character History” function [13].

### Statistical analysis

Three Kruskal-Wallis rank sum tests [14] were performed in R [15] in order to test the significance of location on rock, cover type, and the combination of these two on total percentage cover in the field surveys. These tests were used in lieu of a two-way Analysis of Variance (ANOVA) because the field data did not fit the assumptions of normality required by parametric tests.

For the experimental data, a repeated measures ANOVA was conducted in R using the lme4 package [16] to test the significance of light condition and cover type on percentage cover of the urchins. Final percent cover (percent cover at the end of 30 minutes) was compared in each of the two light conditions using an unpaired t-test [14] to determine if there was a significant difference between final percentage of red cover and clear cover in each light condition.

A series of Friedman rank sum tests [14] were run to test the significance of cover type, light condition, and the combination of the two on total percent cover of the urchins after 50% spine removal. The Friedman test was necessary because the data did not fit the requirements of the parametric equivalent. Individual was used as the blocking variable as each urchin was tested more than one time. Final percent cover of each cover type in each light condition was compared using a series of Wilcoxon rank sum tests [14], and final percent cover of the same cover type was compared for both light conditions to what was seen prior to spine loss using sign tests for related samples [14]. The ggplot2 package in R [17] was used to produce all graphs.

## Results

### Field Study

**Fig 3:**
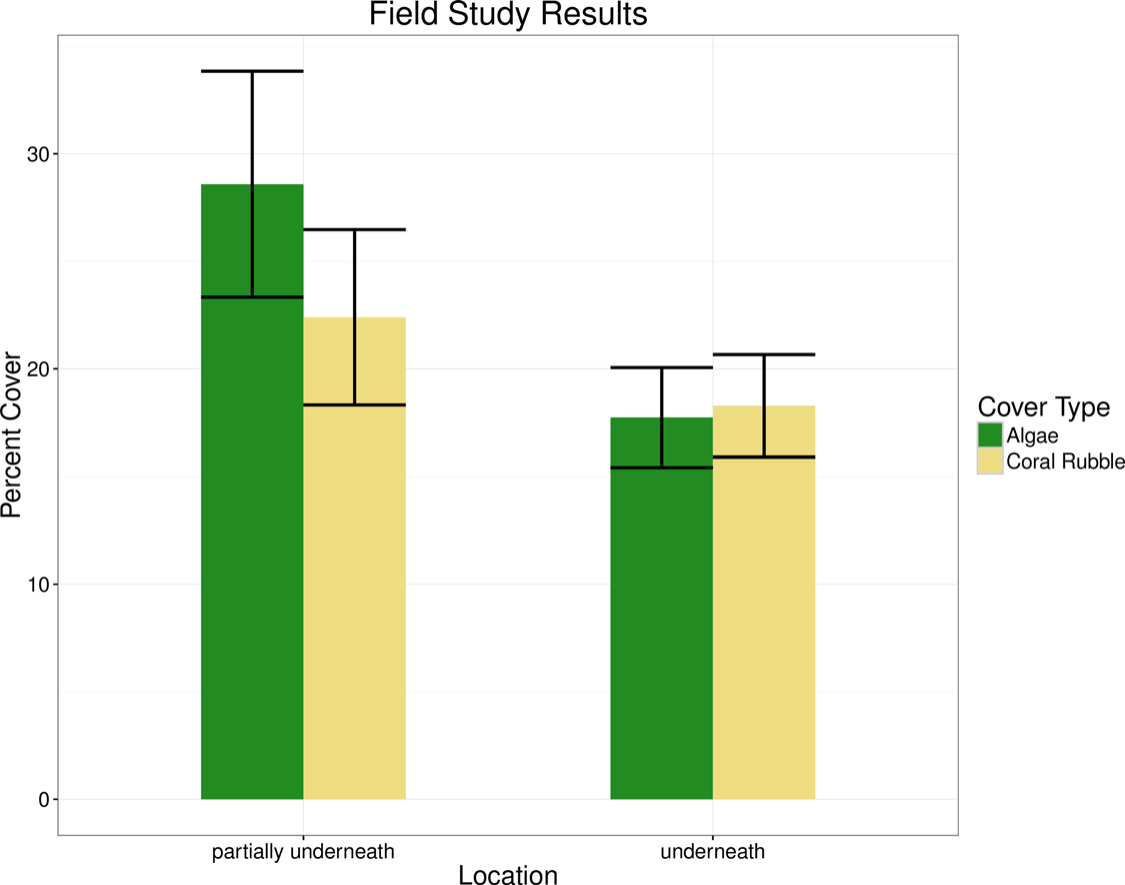
Urchin cover in the field. Percent cover of algae and coral rubble on urchins in found partially underneath or totally underneath rocks in the motu Tiahura reef.

In the field, urchins partially underneath rocks covered themselves with algae and coral rubble more than urchins totally underneath rocks (25.6 and 18.0 percent cover, respectively)(Fig 3). This difference of 7.6 percent was statistically significant (Kruskal-Wallis rank sum test, chi-squared = 10.571, *P* < 0.01). In terms of cover type, there was a minor overall preference of algae over coral rubble, but this was not statistically significant (Kruskal-Wallis rank sum test, chi-squared = 0.087, *P* > 0.05) (Fig 3). However, urchins partially underneath rocks did prefer algal cover over coral rubble cover, with a total percentage of algal cover of 28.6 percent versus a total coral rubble cover of 22.4 percent, a difference of 6.2 percent (Fig 3). This interactive effect between location and cover type was statistically significant (Kruskal-Wallis rank sum test, chi-squared = 12.476, *P* <0.01).

### Experimental Study

#### Pre-spine removal

**Fig 4:**
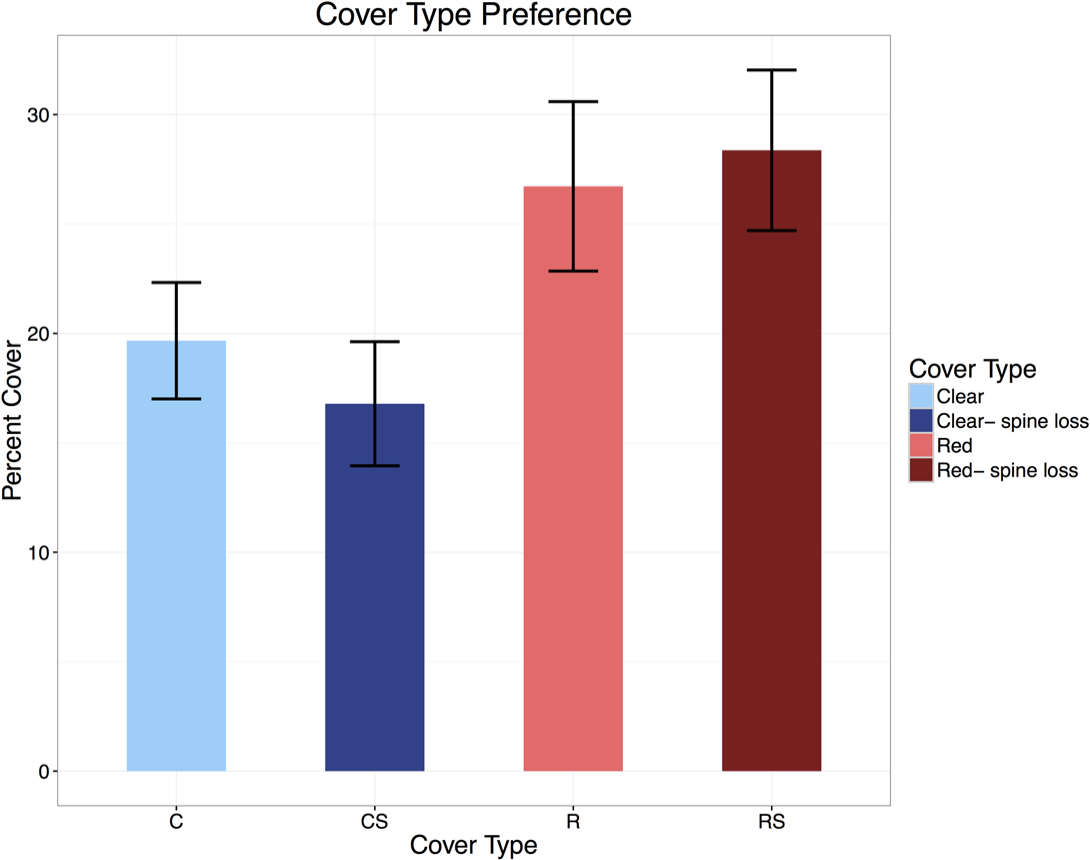
Average cover pre and post spine loss. Average percent cover of both cover types comparing pre and post spine loss data. Both light conditions and all individuals were averaged into one column for each cover type in order to better visualize the differences in covering pre and post spine loss. Cover percentages were taken at the end of the thirty -minute test period.

The total final percent cover (both cover types added together at the end of 30 minutes) of plastic was 54.9 percent in the bright condition, and 38.6 percent in the dim condition. This difference of 16.3 percent was statistically significant (repeated measures ANOVA, *T*_2775_ = 2.509, P < 0.05) (Fig 4).

**Fig 5:**
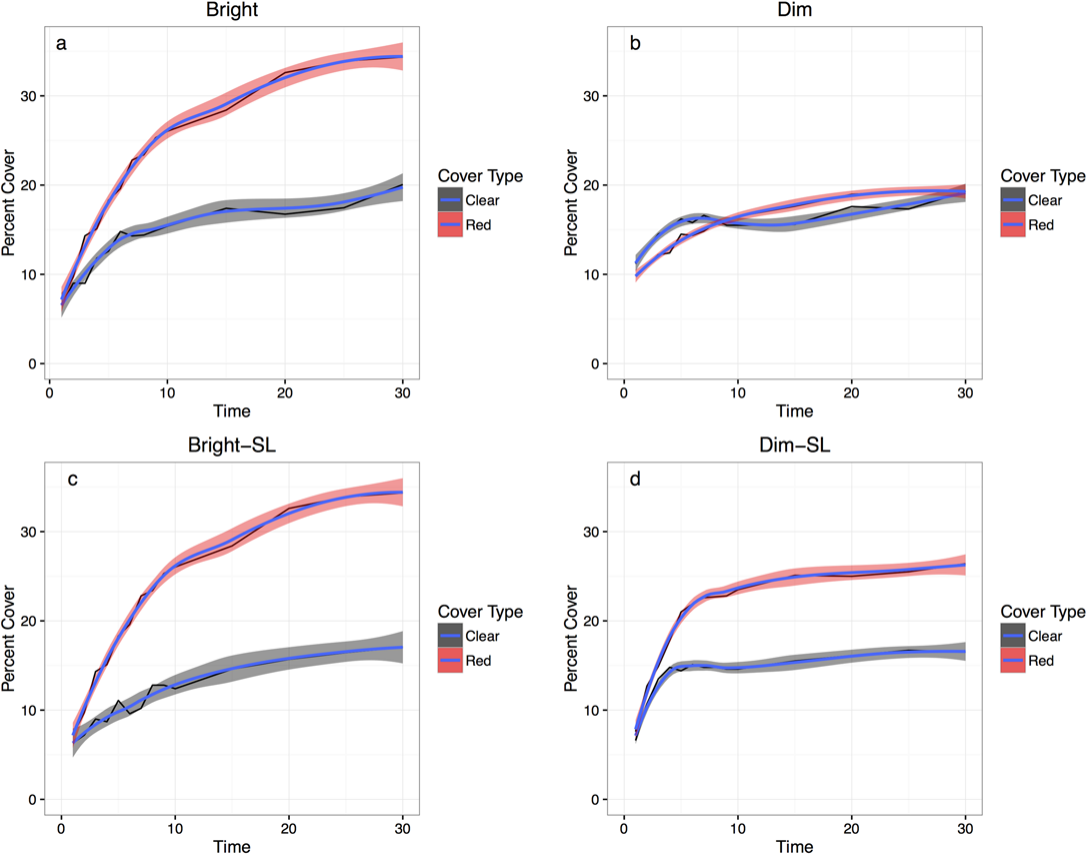
Average percent cover versus time. Average values of percent cover versus time in the bright light condition pre-spine loss (5a), in the dim light condition pre-spine loss (5b), in the bright light condition post-spine loss (5c), and in the dim light condition post-spine loss (5d). ‘Red’ refers to opaque plastic cover, and ‘clear’ refers to clear plastic cover. Lines of best fit, calculated from the geom_smooth function in R as conditional means, are included in blue, and error margins are represented by shaded areas.

The average percentage of red (opaque) cover at the end of thirty minutes in bright light conditions was 34.8 percent, while that of clear cover in the same condition was 20.1 percent (Fig 5A), and this difference of 14.7 percent was statistically significant (Welch two sample t-test, *T*_76.244_ = 4.1442, *P* < 0.0001). In the dim light conditions (Fig 5B), the average percentage clear cover and red cover at the end of thirty minutes were both 19.3 percent, and so no statistically significant difference existed between them (Welch two sample t-test, *T*_*97*.*406*_ = 0, *P* > 0.05).

#### Post-spine removal

For the tests conducted post-spine removal, total final percent cover (red and clear cover at the end of 30 minutes) was 16.1 percent in the bright conditions, and 17.4 percent in the dim conditions. This difference of 1.3 percent was not statistically significant (Friedman rank sum test, chi-squared = 0.4, *P* > 0.05) (Fig 4).

On average, percentage cover of red plastic increased after spines were removed, while percentage cover of clear plastic decreased, but these differences were slight (Fig 4). The average percent cover at the end of thirty minutes was 30.1 percent for red plastic and 17.4 percent for clear plastic in the bright light condition (Fig 5C). In the dim light condition, the average percent cover at the end of thirty minutes was 26.4 percent for red cover and 16.0 percent for clear cover (Fig 5D). These differences between red and clear plastic (12.7 and 10.4 percent, respectively) were both statistically significant (Wilcoxon rank sum tests, W = 1535, P < 0.01, W = 1647, P < 0.01). The difference between the average final red cover percentages (pre and post spine loss) in the bright condition was 4.7 percent and in the dim condition was 7.1 percent. The difference between the average final clear cover percentages (pre and post spine loss) was 2.7 percent in the bright condition and 3.3 percent in the dim condition. However, none of these four differences in slope (ie, difference of red/clear cover slope in the bright/dim light conditions before and after spine loss) was statistically significant (sign tests, *T* = 21, *P* > 0.05, *T* = 15, *P* > 0.05, *T* = 24, *P* > 0.05, *T* = 18, *P* > 0.05).

### Phylogeny

Covering behavior was successfully mapped onto a pre-existing phylogeny courtesy of Littlewood and Smith (1995) (S1 Fig). Covering behavior was found in the genera *Glyptocidaris* and *Stronglyocentrotus* [18], *Temnopleurus* [19], *Mespilia* [20], Salmacis [21], *Echinus* [22], *Psammechinus* [23], *Paracentrotus* [24], *Sphaerechinus* [25], and *Lytechinus* [26]. The behavior evolved once, and was lost in the genera *Glyptocyphus* [27], *Colobocentrotus* [28], and *Heliocidaris* [29] (S1 Fig). The other genera included in the phylogeny did not exhibit covering behavior. Covering behavior was also found in other genera within the order Camarodonta that were not included in the phylogeny. These genera were *Pseudoboletia* [30], *Toxopneustes* [31], *Pseudechinus* [32], and *Genocidaris* [33].

## Discussion

The results of the field study indicated that urchins partially underneath rocks covered themselves more using algae and rock/shells than urchins totally underneath rocks. This supported the hypothesis that urchins cover themselves to protect from sunlight, as urchins that are only partially under rocks are more vulnerable to light than urchins totally underneath rocks. However, this did not rule out other covering explanations such as camouflage from predators [34], or protection from strong currents [7, 31].

A previous study of *T*. *gratilla* has suggested that urchin cover is random with respect to the environment [7]. However, in the present study a link was found between cover type and location in that partially exposed urchins had more cover than urchins fully underneath rocks. Additionally, partially exposed urchins had more algal cover than coral cover. This result refuted the hypothesis that urchins cover to weigh themselves down, as in this case heavier coral cover, which can be more closely held to the test, would be more advantageous than algae to urchins that are more exposed [31]. However, this study did not quantify whether coral rubble and algal cover were equally available in the environment, so it cannot be said what percentage of algae and what percentage of coral could have been considered a true random sample of the environment. Additonally, this study did not qualify whether coral was more or less opaque than algae, or what the significance of such a difference might be.

Urchins subjected to bright light did prefer cover that protected them from the sun, and in dim conditions had no significant preference in cover type. If covering behavior was mainly influenced by another factor, such as predators or currents, no significant difference should have been seen between the two light conditions. This result added further support to the hypothesis that urchin covering in *T*. *gratilla* is a response to sunlight [35, 36, 37].

Spine loss did not limit urchins’ ability to detect, or use, available plastic cover. Based on this result, it seems that tube feet, not spines, are the crucial factor in covering ability for *T*. *gratilla* urchins. However, covering behavior was altered by spine removal in that bright light was no longer correlated with the percent of urchin test covered by plastic. The lack of difference between total percentage of urchin cover in the bright and dim conditions post-spine loss refuted the idea that light is the only trigger for covering behavior, in which case spine loss would have had no effect. The similarity in covering percentages in both light conditions post-spine loss suggests that covering may also be related to general protection. Once spines were removed, urchins may have covered themselves more to shield their bodies from environmental and predatory threats, to which they were now more vulnerable. This result supported the hypothesis that urchin covering in some species is related to protection from predators [37].

These results suggest that *T*. *gratilla* preferred red cover to clear cover, which was evidence of the ability of *T*. *gratilla* to sense the better (more opaque) cover type using their phototaxic tube feet [9] even when the light was not bright. It adds further support to the idea that covering is related to light intensity because there was not another reason for urchins to prefer the opaque cover, as both plastics were very similar. This result also supported the hypothesis that spine loss would not have an effect on covering material preference.

From the phylogeny (S1 Fig) it appeared that covering behavior evolved in sea urchins in the common ancestor of *Glyptocidaris* and its sister group. This behavior was subsequently lost twice: once in the genus *Glyptocyphus* and again in the Colobocentrotus-Heliocentrotus clade, possibly because these clades evolved other protection methods and no longer needed to cover themselves for protection.

## Conclusion

Overall, the results of this study support the hypothesis that covering behavior in *T*. *gratilla* urchins is heavily influenced by light condition, and that a primary motivation for urchin covering behavior is protection from sunlight. Though in the field, preference for covering materials was not statistically significant on its own, the experimental study showed that *T*. *gratilla* covered itself more under bright light and had a strong preference for opaque cover, a result that has not been previously noted for this species. Spine loss does affect urchin behavior by making urchins more likely to cover themselves, perhaps as a way of compensating for the loss of spines that protect them from predators. However, the more opaque cover was preferred in both light and dim conditions. Additionally, though spine loss does impede movement, it does not impede covering behavior. This suggests that urchins with lost spines can still successfully protect themselves from predators via covering, which has been shown to reduce predation over short periods of time [37]. The results add support for the link between *T*. *gratilla* covering behavior and UV radiation, and also suggest that injured *T*. *gratilla* might cover more to protect themselves from predation or injury.

Covering behavior is related to light intensity, and this insight may inform our understanding of the phylogeny of this behavior (S1 Fig).

## Supporting Information

S1 Fig Covering Behavior Phylogeny. Phylogeny of urchin species via Littlewood and Smith (1995), modified in Mesquite. Covering behavior is noted in black.
(TIFF)
