# Supplementary figures and images for: Best Dressed Test: A Study of the Covering Behavior of the Collector Urchin Tripneustes gratilla
Source: PLoS One. 2016 Apr 13;11(4):e0153581. doi: 10.1371/journal.pone.0153581 (PMC4830529; doi:10.1371/journal.pone.0153581)

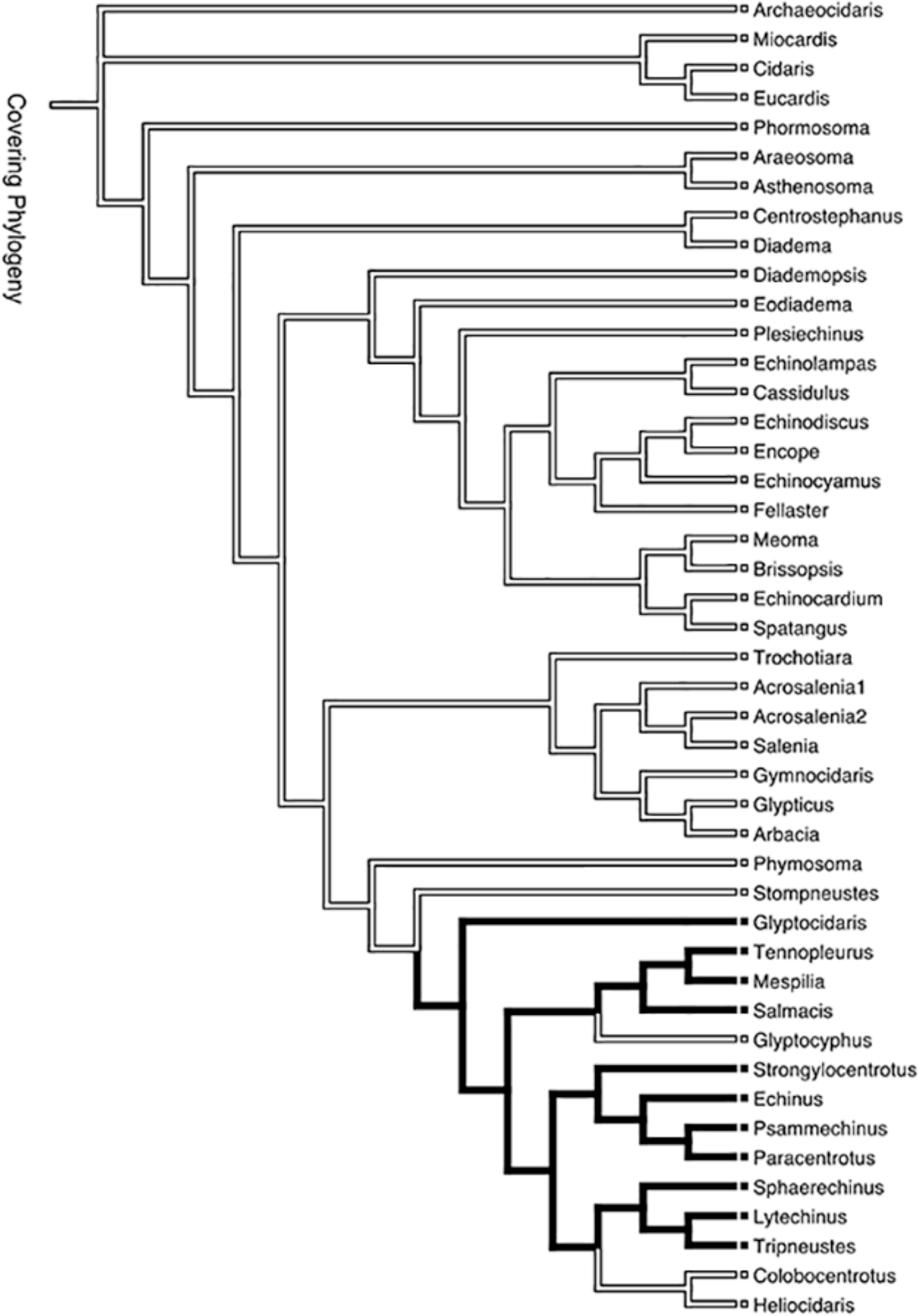

Supplement: S1 Fig — Phylogeny of urchin species via Littlewood and Smith (1995), modified in Mesquite. Covering behavior is noted in black. (TIFF) [file pone.0153581.s001.tiff]
